# Reappraisal of the outcome of healthcare-associated and community-acquired bacteramia: a prospective cohort study

**DOI:** 10.1186/1471-2334-13-344

**Published:** 2013-07-24

**Authors:** Pilar Retamar, María Dolores López-Prieto, Clara Nátera, Marina de Cueto, Enrique Nuño, Marta Herrero, Fernando Fernández-Sánchez, Angel Muñoz, Francisco Téllez, Berta Becerril, Ana García-Tapia, Inmaculada Carazo, Raquel Moya, Juan E Corzo, Laura León, Leopoldo Muñoz, Jesús Rodríguez-Baño

**Affiliations:** 1 Unidad de Enfermedades Infecciosas y Microbiología, Hospital Universitario Virgen Macarena, Avda Dr Fedriani 3, 41009 Seville, Spain; 2 Unidad Clínica de Microbiología y Enfermedades Infecciosas, Hospital del SAS, Jerez de la Frontera, Cádiz, Spain; 3 Sección Enfermedades Infecciosas, Hospital Universitario Reina Sofía, Córdoba, Spain; 4 Unidad Clínica de Enfermedades Infecciosas y Microbiología, Hospital Universitario Virgen de la Victoria, Málaga, Spain; 5 Unidad Clínica de Enfermedades Infecciosas, Microbiología y Medicina Preventiva, Hospital Universitario Virgen del Rocío, Sevilla, Spain; 6 Servicio de Microbiología, Hospital Costa del Sol, Marbella, Málaga, Spain; 7 Servicio de Medicina Interna, Hospital de la Serranía, Ronda, Málaga, Spain; 8 Unidad de Enfermedades Infecciosas, Hospital de La Línea, Cádiz, Spain; 9 Unidad Clínica de Enfermedades Infecciosas y Microbiología, Hospital Punta de Europa, Algeciras, Cádiz, Spain; 10 Servicio de Microbiología, Hospital Puerta del Mar, Cádiz, Spain; 11 Servicio de Microbiología, Complejo Hospitalario de Jaén, Jaén, Spain; 12 Servicio de Medicina Interna, Hospital de Antequera, Málaga, Spain; 13 Unidad Clínica de Enfermedades Infecciosas y Microbiología, Hospital Universitario de Valme, Sevilla, Spain; 14 Servicio de Enfermedades Infecciosas, Hospital Torrecárdenas, Almería, Spain; 15 Unidad de Enfermedades Infecciosas, Hospital Universitario San Cecilio, Granada, Spain; 16 Department of Medicine, University of Seville, Seville, Spain

**Keywords:** Bloodstream infections, Bacteremia, Community-acquired, Healthcare-associated, Antimicrobial therapy, Mortality, Outcome, Antimicrobial resistance

## Abstract

**Background:**

Healthcare-associated (HCA) bloodstream infections (BSI) have been associated with worse outcomes, in terms of higher frequencies of antibiotic-resistant microorganisms and inappropriate therapy than strict community-acquired (CA) BSI. Recent changes in the epidemiology of community (CO)-BSI and treatment protocols may have modified this association. The objective of this study was to analyse the etiology, therapy and outcomes for CA and HCA BSI in our area.

**Methods:**

A prospective multicentre cohort including all CO-BSI episodes in adult patients was performed over a 3-month period in 2006–2007. Outcome variables were mortality and inappropriate empirical therapy. Adjusted analyses were performed by logistic regression.

**Results:**

341 episodes of CO-BSI were included in the study. Acquisition was HCA in 56% (192 episodes) of them. Inappropriate empirical therapy was administered in 16.7% (57 episodes). All-cause mortality was 16.4% (56 patients) at day 14 and 20% (71 patients) at day 30. After controlling for age, Charlson index, source, etiology, presentation with severe sepsis or shock and inappropriate empirical treatment, acquisition type was not associated with an increase in 14-day or 30-day mortality. Only an stratified analysis of 14th-day mortality for Gram negatives BSI showed a statically significant difference (7% in CA vs 17% in HCA, p = 0,05). Factors independently related to inadequate empirical treatment in the community were: catheter source, cancer, and previous antimicrobial use; no association with HCA acquisition was found.

**Conclusion:**

HCA acquisition in our cohort was not a predictor for either inappropriate empirical treatment or increased mortality. These results might reflect recent changes in therapeutic protocols and epidemiological changes in community pathogens. Further studies should focus on recognising CA BSI due to resistant organisms facilitating an early and adequate treatment in patients with CA resistant BSI.

## Background

Bloodstream infections (BSI) remain a leading cause of morbidity and mortality [1]. The early administration of appropriate antibiotics, which has been shown to be an independent protector for mortality in many analyses [2,3], is crucial to improving the outcome for patients with BSI. Epidemiological data are essential for designing treatment protocols adapted to the local area that comprise coverage against the most prevalent organisms, including those producing emerging antimicrobial resistance mechanisms.

In the last decade, a new acquisition category, healthcare-associated (HCA) infections, has been proposed as a result of the development of ambulatory alternatives to hospitalized healthcare [4-10]. Previous studies associated HCA BSI with an increased risk for drug-resistant organisms, inappropriate empirical therapy, and mortality, when compared to strict community-acquired (CA) episodes [4,6,8,9,11]. However, since these data were reported, various multidrug-resistant (MDR) organisms have emerged as a cause of strict community-acquired BSI (mainly extended-spectrum beta-lactamase [ESBL]-producing enterobacteriaceae and methicillin-resistant *Staphylococcus aureus*) [12,13]. Additionally, in Spain, recommendations for empirical therapy were changed in order to consider both HCA organisms and multidrug-resistant community-borne organisms in specific situations [12,14]. The increased awareness that CO- BSI may be caused by specific antibiotic-resistant organisms would have led to the reduced risk of these patients receiving inappropriate therapy. In this context, the objective of our study was to analyse the current etiology, therapy and outcomes for strict community-acquired (CA) and HCA bacteremia in our area.

## Methods

The strengthening the reporting of observational studies in epidemiology (STROBE) recommendations for reporting observational studies [15] were followed in this report.

### Study design and patient selection

This analysis is part of the SAEI/SAMPAC Bacteremia project, aimed at investigating different aspects of BSI and improving the management of patients with BSI in Andalusia, Spain. The study involved a prospective cohort of all consecutive adult in-patients with clinically significant BSI, in 15 public hospitals (10 tertiary and 5 community) in Andalusia, Spain, from October 1rst to December 31th 2006 (to March 31th 2007 in community centres). Patients were followed for 30 days. In this report, we analysed 341 episodes of CO-BSI in the cohort (192 [56%] classified as HCA, and the remainder as CA). Patients with community-onset bacteremia not admitted to hospital were not included in this study (those were 1 CA and 3 HCA episodes from the initial cohort). The sample size was similar to that in previous studies [4,8,9], which would provide a β error of 0.71 for a 10% crude difference in mortality between HCA and CA episodes. The general epidemiological data for HCA and CA episodes have been reported previously [10], being the main significant differences that the CA presented more often an urinary source of the BSI (31% vs 21%, p = 0,04) and were more often caused by *S. pneumoniae* (18% vs 6%, p = 0,001); the HCA BSIs were more often developed by neutropenic patients (1% vs 7%, p = 0,02), more often related to a previous antimicrobial use (18% vs 32%, p = 0,003), the source was more often unknown (15% vs 23%, p = 0,05) or secondary to a catheter device (0% vs 12%, p < 0,001) and was more often caused by *P. aeruginosa* (1% vs 9%, p = 0,01). As previously reported, differences in mortality between HCA and CA BSI were not statistically significant, either at day 14 (18% vs 15%, p = 0.47) or at day 30 (21% vs 19%, p = 0.67) [10].

Susceptibility results were interpreted according to the Clinical Laboratory Standards Institute (CLSI) recommendations. ESBL production was confirmed by the microdilution method if a 3 twofold dilution decrease in the MIC of either ceftazidime or cefotaxime tested in combination with clavulanic acid versus the MIC of each agent when tested alone was observed [16].

The study was approved by the Ethics Committee of the Hospital Universitario Virgen Macarena, which waived the need to obtain informed consent.

### Variables and definitions

The BSI was considered to be CO if the blood cultures had been taken during the first 48 hours of hospital admission, unless the infection was considered to have potentially been acquired during a recent hospital admission to an urgent care centre. The episodes were classified as HCA, following Friedman et al. [4], when any of the following was present: intravenous therapy or specialist nursing care at home in the 30 days before the BSI; haemodialysis or intravenous chemotherapy in the 30 days before the BSI; hospitalization for > 2 days in an emergency care hospital in the 90 days before the BSI; or the patient resided in a nursing home or long-term care facility. Episodes with none of the previous features were classified as CA.

Data were obtained from the charts and included: demographics; ward of admission; presence of underlying chronic diseases and severity according to the Charlson index [17]; invasive procedures; antimicrobial use in the preceding 3 months; source of BSI using CDC criteria [18];severity of the illness the day before the onset of bacteremia (day -1) using the Pitt score [19]; severity of systemic inflammatory response syndrome (SIRS at day 0, using predefined criteria [20]; etiology, and treatment adequacy. Empirical therapy was considered appropriate when an active antimicrobial agent (according to susceptibility data) was administered at recommended doses within the first 24 h after the blood cultures had been performed, and inappropriate otherwise. Pathogens were considered multidrug-resistant according to Magiorakos et al’s criteria [21]. As outcome variables, we used all-cause mortality at days 14 and 30, and inappropriate empirical therapy.

### Statistical analysis

Univariate analysis was performed using the chi-squared or Fisher’s exact test, and the Student’s *t*-test or Mann–Whitney *U*-test, for comparison of categorical and continuous variables, respectively. Crude association between exposure to different variables and mortality or inadequate treatment was estimated by calculating crude relative risk (RR), with 95% confidence intervals (CI). Multivariate analyses were performed using logistic regression. Any variable in the univariate analysis related to mortality at a conservative significance level of < 0.2 was included in the initial model. Variables were selected using a backward stepwise process. Interactions between exposure to the variable of interest and other variables were investigated. Since HCA acquisition was our exposure variable of interest, it was retained in the final models. The validity of the models was evaluated using the Hosmer-Lemeshow test for estimating goodness of fit to the data, and its discrimination ability by the area under the Receiver Operating Characteristics (ROC) curve. All analyses were carried out using the SPSS 15.0 software package (SPSS Inc., Chicago, IL, USA).

## Results

### Mortality predictors in community acquired BSI

The crude analysis for the association between exposure to qualitative variables and 14- and 30-day mortality rates is shown in Table 1. Neutropenia, ICU admission, presentation with severe sepsis or shock, and BSI due to *Pseudomonas aeruginosa* were associated with significant increased 14- and 30-day mortality. A stratified analysis of mortality for Gram-positive and Gram-negative BSI comparing HCA and CA BSI showed a statically significant difference in 14th- day mortality among Gram-negative BSI (7% in CA vs 17% in HCA, p = 0,05). Respiratory tract infection as source of infection and inappropriate empirical therapy were associated with 14-day mortality only. With respect to the quantitative variables, age, Charlson index, and Pitt score were significantly higher in patients who died, compared to survivors at both day 14 (p values: 0.05, 0.002 and < 0.001, respectively) and 30 (p value: 0.05, 0.001, and < 0.001, respectively).

**Table 1:** Univariate analysis of the association of exposure to qualitative variables and 14- and 30-day mortality

**Variable**		**No. dead at day 14 (%)**	**RR(95% CI)**	**P value**	**No. dead at day 30 (%)**	**RR (95% CI)**	**P value**
Gender	Male	34 (17)	Ref.	-	42 (22)	Ref.	-
	Female	22 (15)	0.9 (0.5–1.4)	0.56	27 (18)	0.9 (0.6–1.3)	0.4
Type of acquisition	Community	22 (15)	Ref	-	29 (19)	Ref.	-
	HCA	34 (18)	1.2 (0.7–2.0)	0.47	41 (21)	1.1 (0.7–1.7)	0.7
Type of hospital	Tertiary	38 (15)	Ref.	-	51 (20)	Ref.	-
	Community	18 (21)	1.4 (0.8–2.3)	0.2	19 (22)	1.1 (0.7–1.7)	0.7
Neutropenia	No	49 (15)	Ref.	-	61 (19)	Ref.	-
	Yes	7 (47)	3.1 (1.7–5.6)	0.001	9 (60)	3.2 (2.0–5.1)	<0.001
ICU admission	No	46 (14)	Ref.	-	61 (13)	Ref.	-
	Yes	10 (45.5)	3.1 (1.9–5.4)	<0.001	9 (41)	2.1 (1.2–3.7)	0.01
Severity of SISR	Sepsis	19 (7.8)	Ref.	-	325 (13.1)	Ref.	-
	Severe sepsis or shock	37 (38.5)	5.0 (3.0-8.3)	<0.001	38/54 (39.6)	3.03 (2.0–4.5)	<0.001
Source of bacteremia	Vascular catheter	1 (4)	Ref.	-	2 (8)	Ref.	-
	Urinary tract	7 (8)	1.9 (0.2–14.9)	0.5	9 (10)	1.3 (0.2–9.2)	1
	Intraabdominal	12 (18)	4.1 (0.6–29.6)	0.1	17 (24)	2.9 (0.7–11.5)	0.1
	Other source	9 (21)	4.9 (0.7–36.5)	0.1	10 (23)	2.7 (0.7–11.4)	0.2
	Unknown	15 (23)	5.5 (0.8–39.7)	0.1	19 (29)	4.5 (0.9–30.1)	0.1
	Respiratory tract	12 (25)	6.0 (0.8–43.5)	0.05	13 (27)	3.2 (0.8–13.2)	0.1
Etiology	CONS	1 (4)	Ref.	-	2 (8)	Ref.	-
	*K. pneumoniae*	2 (8)	2.0 (0.2–20.6)	1	4/(17)	2.0 (0.4-)	0.7
	*E. coli*	13 (10.5)	2.5 (0.3–18.3)	0.5	17 (14)	1.7 (0.4–6.7)	0.7
	*S. aureus*	7 (22)	5.2 (0.7–39.9)	0.1	8 (25)	3.0 (0.7-12.9)	0.2
	*S. pneumoniae*	8 (22)	5.3 (0.7–39.9)	0.1	10 (28)	3.3 (0.8–13.9)	0.1
	*Enterococcus spp.*	3 (23)	5.5 (0.7–48.0)	0.1	3 (23)	2.8 (0.5–14.5)	0.2
	*Enterobacter spp*	2 (22)	5.3 (0.5–1.9)	0.2	3 (33)	4.0 (0.8–20.1)	0.1
	*P. aeruginosa*	5 (36)	8.6 (1.1–66.1)	0.02	6 (43)	5.1 (1.2–22.1)	0.03
Empirical therapy	Adequate	40 (14)	Ref.	-	53 (19)	Ref.	-
	Inadequate	16 (28)	2.0 (1.2–3.3)	0.01	17 (30)	1.6 (1.0–2.5)	0.1

*RR* relative risk, *CI* confidence interval, *Ref* reference, *HCA* healthcare-associated, *SIRS* systemic inflammatory response syndrome, *ICU* intensive care unit, *CONS* coagulase-negative staphylococci.

A multivariate analysis of variables associated with 14-day and 30-day mortality was performed next. Variables introduced were: age, HCA acquisition, ICU admission, type of hospital, Charlson index, neutropenia, Pitt score, presentation with severe sepsis or shock, major sources of infection, most frequent pathogens and inappropriate empirical therapy. The interactions between empirical therapy and source, aetiology and severity of SIRS were also studied. HCA acquisition was not associated with an increased risk of death at days 14 or 30. The final models are shown in Table 2. P values for the Hosmer-Lemeshow goodness of fit test for models obtained for 14- and 30-day mortality were 0.99 and 0.84, and the areas under the ROC curve were 0.86 and 0.82, respectively, showing a high predictive ability. Removing HCA-acquisition from the models did not change predictive ability.

**Table 2:** Multivariate analysis of variables associated with mortality in patients with community-onset bloodstream infections

	**β coefficient**	**OR (95% CI)**	**P value**
**Mortality at day 14**			
Healthcare-associated bacteremia	0.06	1.06 (0.52–2.14)	0.86
Charlson index	0.32	1.37 (1.16–1.63)	<0.001
Presentation with severe sepsis or shock	1.74	5.70 (2.13–15.23)	0.001
Urinary tract source	-1.85	0.15 (0.05–0.44)	<0.001
Inappropriate empirical therapy	1.18	3.33 (1.42-7.69)	0.005
**Mortality at day 30**			
Healthcare-associated bacteremia	-1.75	0.83 (0.45–1.57)	0.57
Age	0.02	1.02 (1.00–1.04)	0.01
Charlson index	0.34	1.41 (1.22–1.63)	<0.001
Pitt score ≥ 2	0.16	1.18- (0.99–1.41)	0.06
Presentation with severe sepsis or shock	1.20	3.32 (1.35–8.14)	0.009
Urinary tract source	-1.68	0.18 (0.07–0.45)	<0.001
Inappropriate empirical therapy	0.69	2.00 (0.95-4.34)	0.07

### Antimicrobial resistance among community-acquired and healthcare-related bloodstream infections aetiologies

A comparison of HCA and CA episodes for key antimicrobial resistances of the most relevant BSI-causing bacteria is shown in Table 3. Overall, resistance to any of the antimicrobials considered, except multidrug-resistance, was more frequent among isolates in HCA episodes. Considering specific organisms, only penicillin-resistance in *S. pneumoniae* was significantly more frequent in HCA than in CA episodes. However, it must be noted that there was a tendency for several organisms which are naturally more resistant to certain antibiotics (such as *Enterobacter* spp. or *Pseudomonas aeruginosa*) to be more frequent in HCA.

**Table 3:** Antimicrobial resistance among the most relevant aetiologies of community-onset bloodstream infections in community-acquired and healthcare-related episodes

	**CA episodes**	**HCA episodes**	**P value**
*Escherichia coli*	N = 57	N = 71	
Amoxicillin-clavulanic acid-resistant	9 (15.7)	9 (12.6)	0.61
Cefotaxime-resistant^1^	5 (8.7)	6 (8.4)	0.94
Ciprofloxacin-resistant	14 (24.5)	26 (36.6)	0.14
Gentamycin-resistant	2 (3.5)	5 (7.0)	0.46
Any resistance	15 (26.3)	25 (35.2)	0.33
*Klebsiella* spp.	N = 12	N = 13	
Amoxicillin-clavulanic acid-resistant	2 (16.6)	4 (30.7)	0.64
Cefotaxime-resistant^1^	1 (8.3)	1 (7.6)	1.0
Ciprofloxacin-resistant	0	1 (7.6)	1.0
Gentamycin-resistant	0	2 (15.3)	0.48
Any resistance	2 (16.6)	3 (23.0)	1.0
*Pseudomonas aeruginosa*	N = 1	N = 13	
Ceftazidime-resistant	0	1 (7.6)	-
Imipenem-resistant	0	2 (15.3)	-
Ciprofloxacin-resistant	0	2 (15.3)	-
Amikacin-resistant	0	0	-
*Streptococcus pneumoniae*	N = 27	N = 11	
Penicillin-resistant	2 (7.4)	5 (45.4)	0.01
Erithromycin-resistant	2 (7.4)	1 (9.0)	0.56
Levofloxacin-resistant	0	1 (9.0)	0.28
Any resistance	2 (7.4)	5 (45.4)	0.01
*Staphylococcus aureus*	N = 10	N = 22	
Methicillin-resistant	0	6 (27.2)	0.14
All isolates	N = 159^2^	N = 203^2^	
Any resistance	32 (20.1)	71 (34.9)	0.001
Multi-drug resistance	10 (6.2)	22 (10.8)	0.18

*CA* strict community-acquired episodes, *HCA* healthcare-associated episodes.

^1^ all of them were ESBL producers. ^2^ 10 and 11 episodes, respectively, were polymicrobials.

### Predictive factors of inadequate empiric treatment

We then analysed predictors for receiving inappropriate empirical treatment. Table 4 shows the findings of the univariate analysis for qualitative variables. ICU admission, cancer, ambulatory intravenous therapy, urinary catheter, previous antimicrobial consumption, a BSI source that was unknown, abdominal or catheter-related, BSI due to *P. aeruginosa*, *Enterobacter* spp., *Enterococcus* spp, or coagulase-negative staphylococci were all associated with inappropriate empirical treatment. HCA-acquisition was not associated with it. None of the quantitative variables (age, Charlson index or Pitt score) showed a statistical association. In the multivariate analysis, we found that cancer, previous antimicrobial use, and vascular catheter-related BSI were independent predictors of inappropriate empirical therapy (Table 4). Again, HCA-acquisition was not associated with inappropriate empirical therapy (p = 0.5). For this model, the p value for the Hosmer-Lemeshow goodness of fit test was 0.61, and the area under the ROC curve was 0.63, showing moderate predictive ability.

**Table 4:** Univariate and multivariate analysis of factors related to inappropriate empirical treatment in community-onset BSI

**Variable**		**Univariate analysis**			**Multivariate analysis**		
		**Inappropriate empirical therapy**	**RR (95% CI)**	**P value**	**OR**	**RR (95% CI)**	**P value**
		**No. (%)**					
Gender	Male	33/195 (17)	Ref.	-	-	-	-
	Female	24/144 (17)	1.0 (0.6–1.6)	0.95	-	-	-
Type of acquisition	CA	23/149 (15)	Ref.	-	-	-	-
	HCA	34/190 (18)	1.2 (0.7–1.9)	0.5	-	-	-
Ambulatory IV therapy	No	46/300 (15)	Ref.	-	-	-	-
	Yes	11/39 (28)	1.8 (1.1–3.2)	0.04	-	-	-
Type of hospital	Tertiary	40/253 (16)	Ref.	-	-	-	-
	Community	17/86 (20)	1.25 (0.7–2.1)	0.4	-	-	-
Cancer	No	38/260 (15)	Ref.	-	1,9	1,04-3,78	0,03
	Yes	19/79 (24)	1.65 (1.0 –2.7)	0.05	-	-	-
Urinary catheter	No	45/295 (15)	Ref.	-	-	-	-
	Yes	12/44 (27)	1.8 (1.1–3.1)	0.05	-	-	-
Central vascular Catheter	No	48/304 (16)	Ref.	-	2,0	1,09-3,81	0,02
	Yes	9/35 (26)	1.6 (0.9–3.0)	0.1	-	-	-
Previous antibiotics	No	36/251 (14)	Ref.	-	-	-	-
	Yes	21/87 (24)	1.7 (1.1–2.7)	0.03	-	-	-
Previous surgery	No	53/328 (16)	Ref.	-	2,0	1,04-3,78	0,03
	Yes	4/11 (36)	2.25 (1.0–5.1)	0.08	-	-	-
Neutropenia	No	53/324 (16)	Ref.	-	-	-	-
	Yes	4/15 (27)	1.6 (0.7– 3.9)	0.3	-	-	-
ICU admission	No	50/317 (16)	Ref.	-	-	-	-
	Yes	7/22 (32)	2.0 (1.4–3.9)	0.05	-	-	-
Severity of SIRS	Sepsis	45/244 (18)	Ref.	-	-	-	-
	Severe sepsis or shock	12/95 (13)	0.7 (0.4–1.2)	0.2	-	-	-
Source of BSI	Urinary tract	8/87 (9)	Ref.	-	-	-	-
	Respiratory tract	7/48 (15)	1.6 (0.6–4.1)	0.3	-	-	-
	Other source	6/40 (15)	1.6 (0.6–4.4)	0.3	-	-	-
	Intraabdominal	15/71 (21)	2.3 (1.1–5.1)	0.03	-	-	-
	Unknown	14/65 (21.5)	2.3 (1.1–5.2)	0.03	-	-	-
	Catheter	7/24 (29)	3.2 (1.3–7.9)	0.01	-	-	-
Polymicrobial	No	48/318 (15)	Ref.	-0.001	-	-	-
	Yes	9/21 (43)	2.8 (1.6–5.0)		-	-	-
Etiology	*S. pneumoniae*	3/36 (8)	Ref.	-	-	-	-
	*S. aureus*	3/31 (10)	1.2 (0.2–5.3)	0.8	-	-	-
	*E. coli*	13/123 (11)	1.3 (0.4–1.2)	0.7	-	-	-
	*K. pneumoniae*	4/24 (16)	2.0 (0.5–8.1)	0.3	-	-	-
	*P. aeruginosa*	4/14 (29)	3.4 (0.9–13.4)	0.06	-	-	-
	CNS	9/24 (37)	4.5 (1.4–15.)	0.01	-	-	-
	*Enterococcus spp.*	7/13 (54)	6.5 (2.0–21.3)	<0.001	-	-	-
	*Enterobacter spp*	6/9 (67)	8.0 (2.5–26.0)	<0.001	-	-	-
Multi-drug- resistant bacteria	No	50/307 (16)	Ref.	-	-	-	-
	Yes	7/32 (22)	1.3 (0.7–2.7)	0.4	-	-	-

*RR* relative risk, *CI* confidence interval, *Ref* reference, *ICU* intensive care unit, *CNS* coagulase-negative staphylococci.

### Differences between empirical therapy regimens used in community-acquired and healthcare-related bloodstream infections

We analysed whether there were significant differences between empirical therapy regimens. Overall, the most frequent antimicrobials used in HCA and CA episodes were β-lactam/β-lactam inhibitors (28% vs 41%; p = 0.1), carbapenems (21% vs 14%, p = 0.1), glycopeptides (19% vs 8%, p = 0.003), third-generation cephalosporins (19% vs 32%, p = 0.001), aminoglycosides in combination (13% vs 11%, p = 0.6), and fluoroquinolones (10% vs 13%, p = 0.3), respectively. With respect to the most frequent sources of BSI, HCA episodes of intra-abdominal BSI were less frequently treated with β-lactam/β-lactam inhibitors than CA episodes (44% vs, 72%, p = 0.02) and more frequently with carbapenems (28% vs 6%, p = 0.03); for the combined catheter-related and unknown source episodes (combined, because they both usually present as sepsis with no apparent source), HCA episodes were more frequently treated with glycopeptides (34% vs 9%, p = 0.001) and less frequently with third-generation cephalosporins (15% vs 50%); no differences were found for urinary tract BSI.

Regarding the number of combined treatment with > 1 drug class among CA and HCA BSI there were no differences between them (34,1% vs 34%, p = 0,99). The combination of a cephalosporin plus levofloxacin was more common among CA episodes (29% vs 21% in HCA BSI, p = 0.32) and the combination of a cephalosporin plus vancomycin was more common among HCA BSI (14% among CA vs 23% in HCA, p = 0.49). These differences were not significant.

In Table 5 we have described the aetiology, main resistance and drug of the 57 episodes that received inadequate empirical treatments (23 CA BSI and 34 HCA BSI). 24% were delayed treatments (5, 22% of the inadequate CA BSI treatments and 9, 25% of the inadequate HCA BSI treatments). The main regimes of inadequate treatments were oxacilin resistant Gram-positives treated with betalactams (3, 13% in the CA group and 7, 205 of the inadequate HCA BSI treatments); fluoroquinolones resistant Gram-negatives treated with quinolones (2, 9% and 4, 12% in the inadequate CA and HCA BI treatments respectively). Also 4 episodes due to amoxicillin-clavulanic resistant *E-coli* were treated with it in the CA BSI group and 2 ESBL producers Gram-negatives were treated with ceftriaxone in the HCA BSI subgroup.

**Table 5:** Inadequate empirical antimicrobial treatments regarding the acquisition and the aetiology

**Community acquired (n = 23)**		**Healthcare-related (n = 32)**	
**Aetiology (main R)**	**Empiric treatment**	**Aetiology (main R)**	**Empiric treatment**
*S. aureus*	CTX^1^	*S. aureus* (FqnR)	CTX + LVX^4^
*S. epidermidis*(oxaR)	CTX	*S. aureus* (oxaR)	AMX-CLV
*S. epidermidis*(oxaR)	CTX	*S. epidermidis* (oxaR)	AMX-CLV
*S. epidermidis*(oxaR)	AMX-CLV^2^	*S. epidermidis* (oxaR)	AMX-CLV
*S. pneumoniae*	Delayed	*S. epidermidis* (oxaR)	Delayed
*Strep. viridans*	CIP^3^	*S. epidermidis* (oxaR)	ERT^5^
*Strep. viridans*	CIP	*S. epidermidis* (oxaR)	CIP
*E. faecalis*	CTX	*S. epidermidis* (oxaR)	PIP-TAZ^6^ + AMK^7^
*E. faecalis*	CIP + MET	*S. pneumoniae*	Delayed
*E. faecalis*	Delayed	*S. pneumoniae*	Delayed
*Rhodococcus equi*	CTX	*E. faecium* (ampicilinR)	ERT + AMK
*E. coli* (Amox-clav R)	AMX-CLV	*E. faecalis*	CTX
*E. coli* (Fqn R)	CIP	*E. faecalis*	CTX-AZT^8^
*E. coli* (Amox-clav R)	AMX-CLV	*E. faecalis*	Delayed
*E. coli* (FQ R)	CIP	*Corynebacterium spp.*	Delayed
*E. coli*	Delayed	*E. coli (*ESBL FqnR)	CTX
*E. coli*	Delayed	*E. coli (*ESBL)	CTX
*E. coli* (Amox-clav R)	AMX-CLV	*E. coli (*FQ R)	CIP
*E. coli* (Amox-clav R)	AMX-CLV	*E. coli*	Delayed
*K. pneumoniae* (Amox-clav R)	AMX-CLV	*K. pneumoniae (*ESBL)	Delayed
*Enterobacter spp.*	AMX-CLV	*K. pneumoniae*	VAN^9^
*Citrobacter spp.*	AMX-CLV	*K. pneumoniae*	VAN
*Bacteroides spp.*	Delayed	*Enterobacter spp*. (cefotax FqnR)	CTX + LVX
		*Enterobacter spp.*	CFZ^10^ + VAN
		*Enterobacter spp.*	Delayed
		*Enterobacter spp.* (Pip-tazR)	PIP-TAZ
		*Enterobacter spp.*	Delayed
		*P. aeruginosa* (ceftazi R)	CFZ
		*P. aeruginosa* (ceftaz R)	CFZ + VAN
		*P. aeruginosa* (FQ R)	CTX + LVX
		*P. aeruginosa* (FQ R)	CTX + LVX
		*Acrhomobacter spp*	VAN
		*Candida spp.*	AMX-CLV

^1^*CTX* cefotaxime, ^2^*AMX-CLV* amoxicillin-clavulanic, ^3^*CIP* ciprofloxacin, ^4^*LVX* levofloxacin, ^5^*ERT* ertapenem, ^6^*PIP-TAZ* piperacilin-tazobactam, ^7^*AMK* amikacin, ^8^*AZT* azytromicin, ^9^*VAN* vancomycin, ^10^*CFZ* ceftazidime.

## Discussion

In the last decade, several studies have been published stating the significance of HCA-acquisition to BSI outcome [4-11]. In several of these, the mortality rate of HCA BSI was significantly higher than that of strict community BSI (15–30% vs 10–16%). Our data showed that HCA acquisition was not independently associated with increased mortality in BSI patients. Higher 14-day mortality among HCA as compared to CA was only found by Gram-negative BSI, even though there was no difference in overall mortality of BSI, which may be in part explained by a higher proportion of CNS among HCA BSI.

There are other possible explanations for why we did not find HCA-acquisition to be a risk factor for a worse prognosis.

Firstly, the definition of an HCA BSI was not uniform between the different studies. We used the criteria developed by Friedman et al. [4], which may be appropriate for specialized ambulatory healthcare patterns and epidemiology in the US, but are perhaps less specific to other areas of the world. For instance, in previous publications from the US, *Staphylococcus aureus* was a major cause of bacteremia in HCA BSI groups [4,6,8], and was related to frequent use of a permanent ambulatory venous catheter. However, the major cause of bacteraemia in HCA episodes in our cohort was *E. coli,* as it was in a previous Spanish study [9]. CO-BSI patients with minor urinary tract procedures have been found to be at an increased risk of ESBL-producing *E. coli*[12]. Using Friedman’s criteria, which may be less sensitive for non-catheter-related HCA BSI, many of those episodes might not be considered as HCA. This means that there may be important differences in epidemiology and outcome of HCA BSI, depending on local epidemiology and healthcare practices.

Secondly, the underlying mortality risk for patients with CA episodes may vary between the studies. We only included patients who required hospitalization, while other studies included patients who were not hospitalized [7] and where the risk of death ought, therefore, to be lower. It should be noted that in our study the acute severity of underlying condition and the severity of SIRS at presentation was similar in patients with HCA and CA BSI.

Thirdly, studies used different follow-up periods for measuring mortality; Friedman et al. found higher mortality in HCA-BSI at six months, but not during hospitalization. A recent systematic review recommends that mortality should be assessed at day 30 for BSI studies [2]. We also collected data at day 14 to provide information about early mortality, which might be related more to the direct effect of bacteremia [3].

And fourthly, controlling for confounding is critical to outcome studies of BSI [2]. Only one previous study performed a multivariate analysis to investigate the impact of HCA-acquisition on mortality [8], although the results were not controlled for source or appropriateness of therapy.

As expected, we found that inappropriate empirical treatment was an important predictive factor for a worse outcome in our study. Contrary to the findings of some earlier studies [6,9], we did not find that HCA episodes were associated with a higher probability of receiving inappropriate empirical therapy, which may further explain why HCA episodes were not associated with increased mortality. When we compared our data against those reported by McDonald et al. [6], who used similar definitions, we found that inappropriate empirical therapy was less frequently administered in HCA episodes in our study (18% vs 25%). The fact that carbapenems and glycopeptides were the second and third antibiotic groups used in HCA episodes suggests that physicians were aware of HCA-acquisition as a risk factor for antibiotic-resistant organisms. On the other hand, in our study, inappropriate empirical therapy was more frequent in CA episodes (using Friedman’s criteria) than in the study by McDonald et al. [6], which may reflect higher frequencies of antimicrobial resistance in community isolates, at least in our area, and the need to include other types of healthcare relation—such as urinary tract procedures—or other invasive ambulatory procedures within the definition of an HCA episode. A further problem relates to the fact that some MDR organisms are spread in the community and these might have been the real cause of some strict community-acquired BSI in our cohort. We observed that from the 57 HCA *E. coli* fluoroquinolones resistance was more common than from CA *E. coli* episodes (37% vs 24%, p = 0,14) and that methicillin resistance was more common in HCA than in CA *S. aureus* (27% vs 0%, p = 0,14) but the difference was not significant may be due to under power. By the contrary cefotaxime-resistant *E. coli* caused similar proportions of HCA and CA episodes (9% vs 9%, p = 0,94). ESBL-producing *E. coli* has been well recognized as a cause of community-onset BSI, as explained above. Our results also confirm that community isolates of MRSA were still anecdotal in our area by the time the blood culture were collected [22].

Regarding the inadequate antimicrobial regimes in Table 5 it is remarkable that 26% were considered inadequate due to a delayed treatment. Fluoroquinolones resistant Gram-negatives treated with ciprofloxacine, oxacilin resitant Gram-positives treated with betalactams and 4 episodes of amoxicillin-clavulanic resistant *E.coli* were the most important causes of inadequate treatment. Although those resistances seem to be more frecuent among HCA episodes it is remarkable the present of amoxicillin-clavulanic resistant pathogens among CA episodes. By the time this data was collected this drug was a first line option in many urinary and intrabdominal infections guidelines [23,24] as *E.coli* resistance rate to amoxicillin-clavulanic was about 10-15% as seem in several Spanish publications [25,26]. In the last years this rate has increased up to 25%-30% so urinary and intraabdominal guidelines have been modified. The variables independently related with inappropriate empirical treatment in community-onset BSI were: a vascular catheter as a source of BSI; cancer; and previous antimicrobial use. All three variables are known risk factors for certain types of pathogens or resistances that are not typical for strict community pathogens, and they should be taken into account when considering empirical therapy.

Our study has some limitations. The number of included cases is limited, although similar to previous studies dealing with an outcome impact of HCA BSI. For instance, as mentioned above, this fact limited the analysis of individual microorganism resistance. The findings would not be applicable to areas with a different epidemiology of BSI or system of healthcare. Since the study period was short, seasonal changes in the etiology of BSI could not be considered. We did not include non-hospitalized patients in our study, so that our findings do not extend to BSI patients who were not hospitalized.

## Conclusions

HCA-acquisition was not a predictor for either inappropriate empirical treatment or increased mortality in our community-onset cohort of BSI patients. These results might reflect recent changes in therapeutic approaches and epidemiological changes. Further studies should focus on recognising CA BSI due to resistant organisms. That may facilitate an early and adequate treatment in patients with CA resistant BSI and therefore, a better outcome.

## Competing interests

Jesús Rodríguez-Baño has been a member of advisory boards for Merck, Pfizer, Novartis, and Janssen, has served as a speaker for Merck, Pfizer, Novartis, Astra-Zeneca, and Janssen, and has received research support from Novartis. All other authors declare no competing interests.

## Authors’ contributions

JRB and MDLP were the coordinators of this multicentre study. PR prepared and drafted the initial manuscript. JRB revised the manuscript critically with focus on methods. Both PR and JRB performed the statistical analyses and critically revised the interpretation of data. MC, MH, FFS, AGT and IC collected and performed the laboratory analysis. CN, EN, FT, JEC, LL, LM, MH, AM and RM contributed to the project design, collected and organised the clinical data. All authors read and approved the final manuscript.

## Pre-publication history

The pre-publication history for this paper can be accessed here:

http://www.biomedcentral.com/1471-2334/13/344/prepub

## Other authors from the SAEI/SAMPAC Bacteremia Group

Fernando Rodríguez-López (H. Reina Sofía, Córdoba), María V. García (H. Virgen de la Victoria, Málaga), Verónica Fernández-Galán (H. Virgen del rocío, Sevilla), Alfonso del Arco (H. Costa del Sol, Málaga), María J. Pérez-Santos (H. Serranía de Ronda, Málaga), Antonio Sánchez Porto (H. de La Línea, Cádiz), Manuel Torres-Tortosa (H. Punta Europa, Algeciras, Cádiz), Andrés Martín-Aspas (H. Puerta del Mar, Cádiz), Ascensión Arroyo (Complejo Hospitalario de Jaén), Carolina García-Figueras (H. del SAS de Jerez, Cádiz) Federico Acosta (H. de Antequera, Málaga), Carmen Florez (H. de Valme, Sevilla), Petra Navas (H. Torrecárdenas, Almería), Trinidad Escobar-Lara (H. San Cecilio, Granada).
